# Immediate hypersensitivity reaction to intravitreal injection of triamcinolone acetonide following treatment by pars plana vitrectomy: A case report

**DOI:** 10.1097/MD.0000000000032893

**Published:** 2023-02-17

**Authors:** Wei Zhang, Mengyu Liao, Hua Yan

**Affiliations:** a Department of Ophthalmology, Tianjin Medical University General Hospital, Tianjin Key Laboratory of Ocular Trauma, Laboratory of Molecular Ophthalmology, Tianjin Medical University, Tianjin, China; b School of Medicine, Nankai University, Tianjin China; c Tianjin Eye Hospital, Tianjin Key Lab of Ophthalmology and Visual Science, Tianjin Eye Institute, Clinical College of Ophthalmology Tianjin Medical University, Tianjin, China.

**Keywords:** allergy, corticosteroids, filtration, triamcinolone acetonide, vitrectomy

## Abstract

Hypersensitivity reactions to intravitreal injection of triamcinolone acetonide (TA) were very rare and had been infrequently reported. In this study, we reported the clinical features of hypersensitivity reactions of TA, explored its management, and analyze the relative factors.

**Patient concerns, diagnosis, interventions, and outcomes:**

We described a case of an immediate hypersensitivity reaction to intravitreal injection of TA following the treatment by pars plana vitrectomy. A 27-year-old man was reported with an immediate hypersensitivity reaction following an intravitreal injection of TA. 0.1 mL TA (40 mg/mL) was injected into the vitreous cavity and then white punctate edema appeared on the surface of the retina. Three days later, the visual acuity was 20/500 and white punctate edema disappeared on the surface of the retina without specific treatment.

**Conclusions and importance:**

Hypersensitivity reaction must be considered in cases of intravitreal injection of TA that may resolve without specific treatment. The clinical intravitreal injection of TA requires filtration to remove excipients.

## 1. Introduction

Intravitreal injection of triamcinolone acetonide (TA) is a treatment for macular edema, age-related macular degeneration, and inflammatory eye diseases, although most reports on its efficacy are based on case reports and uncontrolled researches. As a crystalline milky liquid, TA remained in the vitreous for a few days after injection, as a discrete white cloud, with little or no reaction from the surrounding vitreous. Therefore, protruding floats are usually encountered after processing, when the material falls from the visual axis, the floats usually disappear within a few days. In this article, we report on hypersensitivity reactions after intravitreal injection of TA.

## 2. Case report

**Figure 1.**
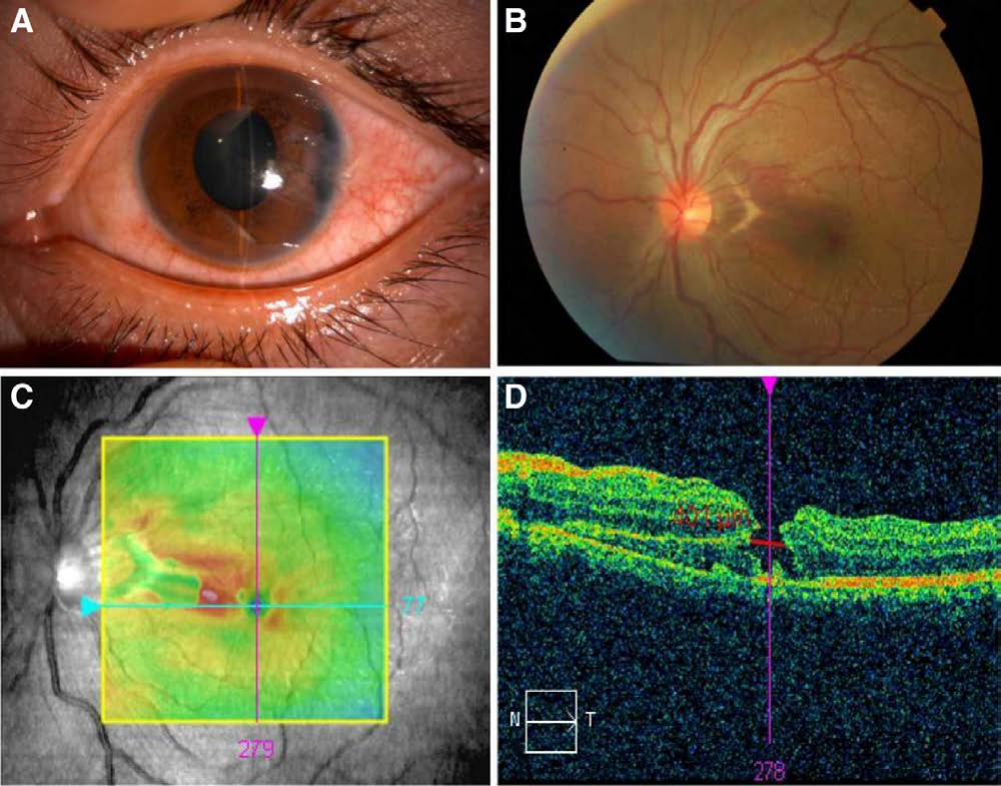
The patient with a history of his left eye being injured from an iron nail 1 month ago. (A) The temporal corneal wound was sutured, the anterior chamber depth was normal, the root of the iris was fractured at 2 to 4 o’clock, and the long diameter of the pupil was about 4 mm. (B) The optic disc boundary of the fundus was clear, choroidal lacerations could be seen on the temporal side of the optic disc. (C) Subretinal hemorrhage could be seen around it. (D) A circular hole with surrounding retinal folds could be seen in the macular area.

A 27-year-old man presented to the Ophthalmology Department of Tianjin Medical University General Hospital in January 2020 with a history of his left eye injured by an iron nail 1 month ago. His left eye visual acuity was 24/200, the temporal corneal wound was sutured, the anterior chamber depth was normal, the root of the iris was fractured at 2 to 4 o’clock, the long diameter of the pupil was about 4 mm (Fig. 1A); the optic disc boundary of the fundus was clear, choroidal lacerations could be seen on the temporal side of the optic disc (Fig. 1B), and subretinal hemorrhage could be seen around it (Fig. 1C), and a circular hole with surrounding retinal folds could be seen in the macular area (Fig. 1D). The size was about 1/4PD. The intraocular pressure was 24 mm Hg.

## 3. Surgical procedure

**Figure 2.**
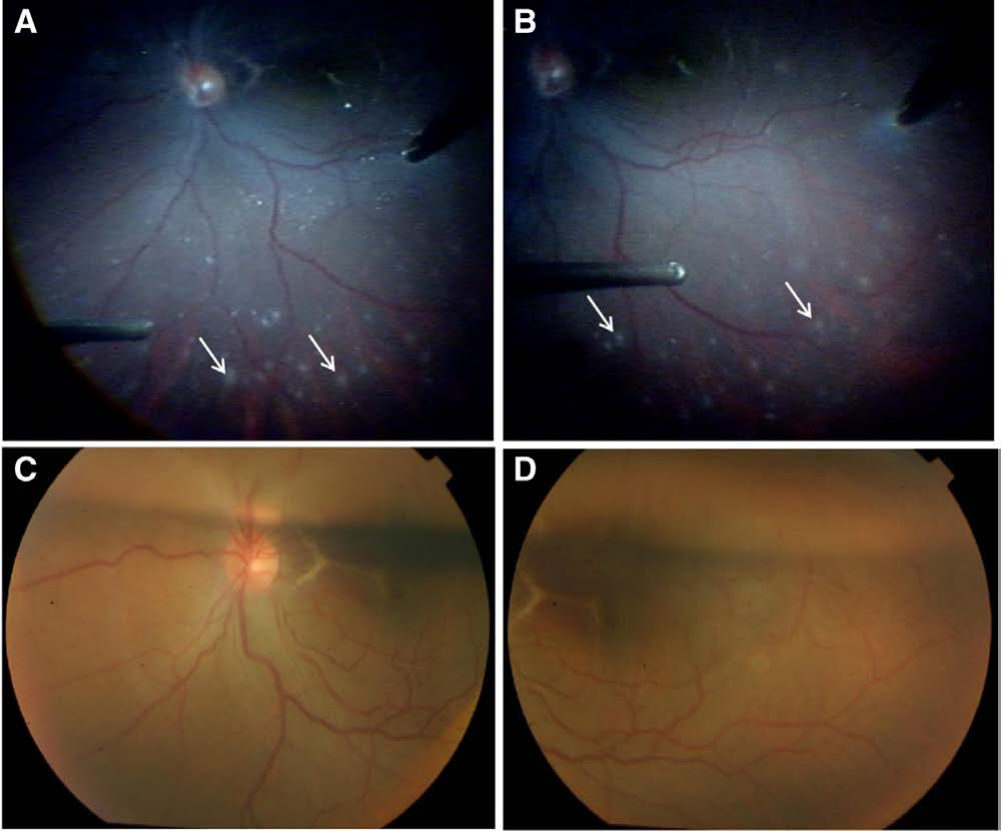
0.1 mL TA (40 mg/mL) was injected into the vitreous cavity which made the residual vitreous gel to be visualized clearly and removed completely. (A–B) During the surgery white punctate edema appeared on the surface of the retina (arrows), which is considered to be an immediate hypersensitivity reaction to TA. (C–D) Three days later, the visual acuity was 20/500 and white punctate edema disappeared on the surface of the retina. The macular hole was closed. TA = triamcinolone acetonide.

One month after the trauma the patient received a vitrectomy. The surgery was performed by Dr Yan. The eye received 2% lidocaine retrobulbar anesthesia and then was prepared for a standard 3-port 23-gauge PPV. Delamination and dissection of the vitreous were performed followed by the clearance of posterior vitreous detachment. Then 0.1 mL TA (40 mg/mL) was injected into the vitreous cavity which made the residual vitreous gel to be visualized clearly and removed completely. During the surgery white punctate edema appeared on the surface of the retina (Fig. 2A and B), which is considered to be an immediate hypersensitivity reaction to TA. Internal limiting membrane peeling was performed to treat the macular hole. After the gas-liquid exchange, air gas was filled in the eye.

## 4. Results

Three days later, the visual acuity was 20/500 and white punctate edema disappeared on the surface of the retina (Fig. 2C and D). The macular hole was closed. The patient reported no pain or tenderness and intraocular pressure was normal.

## 5. Discussion

TA is a glucocorticoid that can cause type I or type IV allergies.^[1]^ TA excipients include 0.75% carboxymethyl cellulose, 0.99% benzyl alcohol (BA), 0.04% polysorbate, isotonic sodium chloride, sodium hydroxide, and hydrochloric acid. The concentration of carboxymethylcellulose is 7.5 mg/mL with high viscosity and no retinal toxicity, but it can cause anaphylactic shock. The concentration of BA is 9 mg/mL and it is a preservative with retinal toxicity and easy-to-cause aseptic endophthalmitis. Several cases of hypersensitivity of TA have been reported, in which the cause was either the corticosteroid^[2]^ or excipients,^[3]^ including BA, carboxymethylcellulose, and polysorbate 80.

In this case, after 0.1 mL TA (40 mg/mL) was injected into the vitreous cavity, white punctate edema appeared on the surface of the retina, which is considered to be an immediate hypersensitivity reaction to TA. Three days later, the visual acuity was 20/500 and white punctate edema disappeared on the surface of the retina. The patient reported no pain or swelling, and the anterior chamber remained quiet. No special treatment was given, and there were no other obvious adverse events. Systemic administration of corticosteroids (oral, parenteral, and intralesional) is a rare cause of immediate or delayed hypersensitivity reactions.^[4]^ The immediate reaction can be clinically manifested as urticaria or rarely allergic reactions.^[5]^

## Author contributions

**Data curation:** Mengyu Liao.

Formal analysis: Mengyu Liao.

Investigation: Wei Zhang, Mengyu Liao.

Methodology: Wei Zhang.

Supervision: Hua Yan.

Validation: Hua Yan.

Visualization: Hua Yan.

Writing – original draft: Wei Zhang.

Writing – review & editing: Hua Yan.
